# Qualitative and Antioxidant Evaluation of High-Moisture Plant-Based Meat Analogs Obtained by Extrusion

**DOI:** 10.3390/foods14172939

**Published:** 2025-08-23

**Authors:** Viorica Bulgaru, Ilkay Sensoy, Natalia Netreba, Angela Gurev, Ulunay Altanlar, Sergiu Paiu, Veronica Dragancea, Rodica Sturza, Aliona Ghendov-Mosanu

**Affiliations:** 1 Faculty of Food Technology, Technical University of Moldova, 9/9 Studentilor St., MD-2045 Chisinau, Moldova; natalia.netreba@tpa.utm.md (N.N.); angela.gurev@chim.utm.md (A.G.); sergiu.paiu@doctorat.utm.md (S.P.); veronica.dragancea@chim.utm.md (V.D.); rodica.sturza@chim.utm.md (R.S.); aliona.mosanu@tpa.utm.md (A.G.-M.); 2 Department of Food Engineering, Middle East Technical University, Universiteler Mahallesi, 06800 Ankara, Türkiye; isensoy@metu.edu.tr (I.S.); ulunay.altanlar@metu.edu.tr (U.A.)

**Keywords:** meat analogs, protein rich plant-based raw materials, protein isolate, extrusion, quality indices, bioavailability

## Abstract

This study investigated meat analogs produced by high-moisture extrusion from mixtures of pea protein isolate and soryz flour, and chickpea flour and hazelnut meal in a 1:1 ratio, at two distinct heating temperature profiles: 40-60-80-100 °C and 60-80-100-120 °C. Physicochemical indicators, texture and chromatic parameters, protein digestibility, and antioxidant activity of the meat analogs were assessed, and antioxidant activity of the product in terms of simulating gastrointestinal digestibility in vitro was performed. The results obtained for the analyzed meat analog indicators were greatly influenced by the type of plant-based raw material used and the heating temperature profiles. A higher temperature regime leads to a slight decrease in the content of nutritive compounds in the final products. All meat analog samples showed good water and oil holding capacity. A decrease in hardness was observed for the mixtures compared to pea protein isolate, which can be attributed to protein content. The digestibility of the processed meat analog proteins ranged between 86.84% and 69.37%. PCA was applied to illustrate the relationships between physicochemical characteristics, protein digestibility, antioxidant activity, texture profile analysis, and CIELab color parameters in high-moisture meat analogs.

## 1. Introduction

The global meat market was valued at $1378 billion in 2023 and was valued at over 350 ×10^9^ kg of meat products per year [1]. According to Rabobank’s quarterly review [2], the meat industry is expected to grow further by 2.5–3% in 2025.

Global demand for meat is projected to increase by 50% over the next two decades, primarily driven by population growth [3,4]. According to a recent analysis from the Food and Agriculture Organisation, 14.5% of the world’s greenhouse gas emissions result from animal farming, and animal agriculture threatens diversity and uses 20% of the world’s freshwater resources [5]. Growing environmental demands have prompted the search for alternative protein sources. Alternatives can be obtained in various ways—high-temperature processing, which converts plant protein into a textured product [6,7], the use of modified strains of bacteria and fungi [8,9], microalgae, insects and single-celled organisms that have high nutritional value and require minimal resources for cultivation [10], and the production of cell-based protein known as cultured meat [11]. Existing methods allow us to obtain products similar in consistency to minced meat [12,13] including burgers and meatballs with textured plant-based protein [14], or tissue engineering.

It is estimated that switching to plant-based substitutes would result in 96–98% less greenhouse gas emissions and 67–97% less freshwater use than switching to all agriculture combined [15].

In recent years, the vegetable protein market has been witnessing a steady growth, with a projected annual growth rate of 8–12%. According to recent market analyses, the global plant protein market was valued at approximately $15.6 billion in 2023 and is projected to reach $23.4 billion by 2028, reflecting a Compound Annual Growth Rate of 8.4%. Overall, the alternative meat sector, derived from various methods, could reach over $140 bn by 2030 [16]. This is due to increasing number of consumers opting for plant-based diets (veganism, flexitarianism).

Consumers are increasingly attentive to the environmental impact of products. The production of plant-based proteins generates significantly fewer greenhouse gas emissions and requires less water and land compared to animal agriculture, making it more appealing to environmentally conscious consumers. Consumer demand patterns for plant proteins as meat analogs are made up of a number of factors including demographic characteristics, customer motivation, eating habits, and the influence of external factors such as marketing or legislative initiatives [17].

However, there is a risk that plant proteins may not fully meet the amino acid requirements as effectively as animal proteins. Cereals are high in starch but relatively low in protein (10–12%), while legumes contain a higher protein content (20–35%). Combining these raw materials can help achieve a well-balanced intake of essential amino acids [18], since legumes are a valuable source of amino acids that are deficient in cereals—such as lysine, methionine, histidine, and aspartic acid—and cereals, in turn, contain higher levels of sulfur-containing amino acids that are less abundant in legumes [19].

On the other hand, plant-based proteins are valued in the production of meat analogs for their functional properties, including water retention, oil uptake, emulsification, and gelation capacity [20,21].

Raw ingredients, whether in the form of flours, extracts, or isolates, are subsequently processed to be transformed into meat analogs [22]. Traditionally, meat analogs were manufactured using simple technologies such as fermentation, chemical-based protein coagulation, pressing, heating, steaming, cooling, and washing [23]. Nowadays, the techniques appreciated in the manufacture of meat analogs are extrusion, shear cell technology, and 3D printing [20], which fit into the objectives of a circular bioeconomy, and are environmentally friendly techniques with an enhancing effect on the quality of the obtained product. During the thermodynamic extrusion process, protein-rich raw materials are transformed into fibrous products within the limits of 50–70%, due to the action of pressure, heat, and mechanical shear [24]. In the extruder, proteins lose their tertiary structure, perhaps even their secondary structure, by crosslinking into a new structure. The crosslinking of protein molecules plays an essential role in the texturing process, allowing the transition of plant proteins from their native globular conformations to fibrous structures, which more closely mimic the organization of animal muscle tissue. Based on the amount of water added during the extrusion process, two types of extrusion are distinguished: low-moisture extrusion (20–40% added moisture) and high-moisture extrusion (40–80% added moisture) [21].

The aim of this study was to develop and analyze the quality characteristics of high-moisture meat analogs produced by combining protein-rich raw materials.

## 2. Materials and Methods

### 2.1. Chemicals

Hydrogen peroxide (30%), *n*-hexane (>95%), hydrochloric acid (38%), potassium bicarbonate (97%), nitric acid ASC reagent (70%), perchloric acid ASC reagent (70%), deionized water, ninhydrin, pepsin (extra pure, powder), tripsin (lyophilized powder), sodium bicarbonate, and trichloroacetic acid (TCA) were obtained from Sigma-Aldrich (Schnelldorf, Germany). Petroleum ether hydroxide, acetone, and n-octan were purchased by Chemapol (Prague, Czech Republic). Kjeldahl mineralization Catalyst CX 5.5 GR was purchased from VWR Chemicals (Lutterworth, UK). vreceiver TKN (Bromocresol green and Methyl red) was obtained from Velp Scientifica (Usmate, Italia). Gallic acid (97%), sodium hydroxide (≥97%), phenolphthalein, and 2,2-azino-bis(3-ethylbenzothiazoline-6-sulphonic acid (ABTS) (≥98%) were acquired from Merck (Darmstadt, Germany). A Specord 200 Plus spectrophotometer (Jena, Germany) was utilized for spectrophotometric measurements.

### 2.2. Materials

For the production of high-moisture meat analog samples, chickpea flour (Vegrano, Kimbiotek Kimyevi Maddeler San. Tic. A.Ş., Istanbul, Türkiye), hazelnut meal (Altaş Yağ Sanayi Ticaret A.Ş., Giresun, Türkiye), pea protein isolate (Vegrano, Kimbiotek Kimyevi Maddeler San. Tic. A.Ş., Istanbul, Türkiye), and soryz flour (soryz variety Alimentar 1, Chisinau, Republic of Moldova,) were utilized.

### 2.3. Manufacture of High Moisture Meat Analog

#### 2.3.1. Technical Properties of Extruder

Extrusion runs were performed using a laboratory scale co-rotating twin-screw extruder (Feza Machine Co., Ltd., Istanbul, Türkiye) with a screw diameter of 25 mm and length to diameter (L/D) ratio of 25:1. A rectangular cooling die was connected to the twin screw extruder using a die adapter. The 360 mm long (7 mm (height) × 25 mm (width)) die had three independently cooled zones, each regulated by water flow.

#### 2.3.2. Production of High Moisture Meat Analogs

Chickpea (C), soryz (S) flours, and hazelnut meal (HM) were blended with pea protein isolate (PPI) in a 1:1 ratio (dry basis). Control samples consisted of pea protein isolate, which did not include any added flour. All formulations were processed at the same moisture level (60%) and subjected to two different temperature profiles. The two distinct barrel heating temperature profiles along the extrusion direction were 40-60-80-100 °C (for this temperature barrel, the samples are marked with the number 1) and 60-80-100-120 °C (for this temperature, barrel the samples are marked with the number 2). Across all samples, the feed flow rate was maintained at 1 kg/h, and the screw speed was set to 215 rpm. The cooling die temperature profile, along with the adapter for the final barrel zone temperature of 100 °C, was set at 100-60-40-30 °C, while for the final zone temperature of 120 °C, it was 120-60-40-30 °C.

After the extrusion process, the products were vacuum-packed using a packaging machine (Packtech, Nurcivan Industrial Packaging Systems Co., Ltd., Istanbul, Türkiye) and stored in a freezer at −18 °C until further analysis.

### 2.4. Meat Analogs Characteristics

#### 2.4.1. Physicochemical Analysis of Meat Analogs

The dry matter (DM) was determined by the oven-drying method using the method AOAC Official Method 925.10. The protein content (PC) was determined according to the Kjeldahl Method in a UDK129 (VELP Scientifica, Usmate, Italy).

Fat content (FC) was determined by the Soxhlet extraction method, using the protocol described in AOAC Official Method 948.22, in a SER148 Solvent Extraction Unit (VELP Scientifica, Monza, Italy).

Ash content (AC) represents the mineral content determined using the dry ashing method. This technique quantifies the inorganic residue remaining after combustion of organic material in a muffle furnace (Omron E5CC, Snol, Lithuania) at 550 ± 1 °C, following the procedure outlined by AOAC (2006).

Crude fiber content (CFC) of the samples was determined using a FIWE Raw Fiber Extractors (VELP Scientifica, Italy), following the procedures described by AOAC (2012).

Carbohydrate content (CHC) of the samples was determined by calculating the difference (100 − (Ash + Protein + TotalFat)), according to [25].

#### 2.4.2. Determination of Meat Analogs pH

To measure the sample pH, a suspension containing 5 g of product in 25 g of distilled water was prepared. The pH was measured with a pH meter SI Analytics, TitroLine 5000 (Weilheim, Germany).

#### 2.4.3. Water/Oil Holding Capacity (WHC/OHC)

In total, 5 g of the analyzed sample was placed in a 50 mL tube with gauze underneath. For each sample, 20 mL of distilled water/oil was added. The samples were mixed and left at room temperature (22 ± 2 °C) for 30 min. The tube was centrifuged at 3000 rpm for 10 min at 35 °C. After centrifugation of the samples, the supernatant was removed and the new mass of the sample was recorded. The water or oil holding capacity was calculated according to Equation (1) [26].

(1) $$WHC/OHC\;\left(mLg^{-1}\right)=\frac{W2}{W1}\times 100$$

where

*W*1—mass of the meat analog before centrifugation, g;

*W*2—mass of the meat analog after centrifugation, g.

#### 2.4.4. Texture Profile Analysis (TPA)

For TPA, the frozen extruded samples were thawed and cut into 24 mm lengths. TPA was performed using a Stable Micro Systems TA.XT plus texture analyzer (Godalming, United Kingdom) equipped with an SMS-P/100 Ø 100 mm probe. Each measurement was carried out in two compression cycles. The TPA test parameters were as follows: load cell capacity, 30 kg; pre-test speed, 1 mm/s; test speed, 0.5 mm/s; post-test speed, 1 mm/s; deformation distance, 3.5 mm (50% strain); waiting time between cycles, 5 s; and trigger force, 50 g. Each sample was analyzed in 12 replicates. Force–time curves were recorded and used to calculate the following parameters. Hardness (N) was defined as the maximum peak force measured during the initial compression. Resilience (%) was calculated as the ratio of the area under the curve during the withdrawal phase of the first compression to the area under the curve during the compression phase, multiplied by 100. Chewiness (N) was calculated as the product of hardness, cohesiveness, and springiness.

#### 2.4.5. CIELab Color Parameters

Color assessments were performed using a colorimeter (Ser-Lab, SL400 Spectrophotometer, Istambul, Türkiye). Color evaluation was carried out for both the raw materials and the extruded products. The raw materials were evenly spread across a solid white surface without any gaps, and color measurements were taken from 15 distinct locations. The frozen extruded samples were initially thawed at room temperature and then sliced into 24 mm lengths. Measurements were taken from 15 extruded pieces. For each piece, the measurement probe was placed on a uniform surface of the sample, ensuring complete contact. The L* (lightness), a* (red-green axis), and b* (yellow-blue axis) values were recorded for each measurement. The extruded product produced with pea protein isolate was used as control, and the color differences (ΔE*) between the mixture samples and the control were calculated using the following formula [27].

(2) $$\Delta E*=\sqrt{{\left(L_{sample}^{*}-L_{control}^{*}\right)}^{2}+{\left(a_{sample}^{*}-a_{control}^{*}\right)}^{2}+{\left(b_{sample}^{*}-b_{control}^{*}\right)}^{2}}$$

Browning index (*BI*) was calculated using the following expression [28]:

(3) $$BI=\left(\frac{\left(a^{*}+1.75\times L^{*}\right)}{\left(5.645\times L^{*}+a^{*}-3.012\times b^{*}\right)}-0.31\right)\times \frac{100}{0.17}$$

Chroma index (C*) indicates the degree of the color and is proportional to the intensity of the color and was calculated according to the formula [29]:

(4) $$C^{*}=\sqrt{a^{*2}+b^{*2}}$$

Hue angle (h*) is expressed in a 0–360° range, where 0° = bluish-red, 90° = yellow, 180° = green and 270° = blue. The h* was calculated using the following expression [28]:

(5) $$h^{*}=arctan\left(\frac{b^{*}}{a^{*}}\right)$$

#### 2.4.6. Digestibility of Proteins

Protein digestibility was expressed in percentage as the ratio between the content of trichloroacetic acid (TCA)-soluble peptides contained in the supernatant obtained in the in vitro digestion and the total protein content of the analyzed samples, according to Equation (6).

(6) $$Protein\;digestibility\;\left(\%\right)=\frac{B}{A}\times 100$$

where

*A*—total protein content, %;

*B*—TCA-soluble peptide content, % [30].

In vitro digestion of meat analog samples was conducted following the INFOGEST 2.0 protocol, which involved a gastric phase with incubation under constant mixing for 2 h at 37 °C and pH 3.0, followed by an intestinal phase lasting 2 h at 37 °C and pH 7.0 [31]. For subsequent analyses, the supernatant without insoluble parts, obtained after centrifugation (17,500 rpm for 10 min), was used. The TCA-soluble peptide content was analyzed and quantified as mg of bovine serum albumin equivalents per kg sample according to [32].

#### 2.4.7. Antioxidant Activity (AA)

The AA of meat analogs was determined for the hydroalcoholic extracts and aqueous extract. The hydroalcoholic extracts were prepared as follows: 1.0 g of sample was mixed with 20 mL of 70% aqueous ethyl alcohol solution (1:20, *m*/*v*), or with 20 mL of distilled water. The mixture was homogenized using a mixer and subjected to ultrasound-assisted extraction (ISOLAB installation, Laborgeräte GmbH, Eschau, Germany) at a frequency of 37 kHz and a temperature of 35 ± 1 °C for 10 min. The extracts were then centrifuged at 12,000 rpm for 25 min. The supernatant was collected, filtered through filter paper, and stored in the refrigerator until further analysis. Aqueous extracts were prepared by combining 1.0 g of sample with 20 mL of distilled water (1:20, *m*/*v*), homogenizing the mixture, and subjected to ultrasound-assisted extraction at the same frequency (37 kHz) at 25 ± 1 °C for 15 min. The pH of the samples was adjusted to 4.3 using 9M HCl, followed by incubation at room temperature for 2 h. Samples were then centrifuged at 12,000 rpm for 25 min. The supernatant was collected, filtered through filter paper, and stored under refrigeration until analysis. The ability to capture ABTS + free radical cations in the TEAC assay was determined according to the method described by Arnao et al. [33]. Results were expressed as mg of trolox equivalents per gram of dry weight (mg TE/g DW).

#### 2.4.8. AA In Vitro Digestion Model

In vitro digestion of meat analog samples was carried out in accordance with the INFOGEST 2.0 protocol [31]. Samples for analysis were collected at the end of the digestion process. The digested mixtures were cooled to 5 °C and centrifuged at 17,500 rpm for 10 min to separate the insoluble material. The resulting supernatants were withdrawn and frozen. The AA of the digested samples was evaluated using the ABTS assay, as described in Section 2.4.7. The results were expressed as mg TE/g DW of the sample [17].

### 2.5. Statistical Analysis

The measurements in this study were performed in triplicate and are reported as mean values and standard error of the mean. Data analysis was conducted using Microsoft Office Excel 2007 (Microsoft, Redmond, WA, USA). Statgraphics, Centurion XVI 16.1.17 (Statgraphics Technologies, Inc., The Plains, VA, USA) program was used for one-way analysis of variance (ANOVA) according to Tukey’s test at a significance level of *p* < 0.05. The Pearson correlation and principal component analysis (PCA) were computed using the Scikit-learn Python library.

## 3. Results and Discussions

### 3.1. Physicochemical Quality Parameters and AA of Meat Analogs

The chemical composition of meat analogs can be very different. This is greatly influenced by the composition of the plant-based raw materials used. Plant-based meat analogs are typically high in protein, low in fat, and varied in moisture content to achieve the desired textural properties and health benefits valued by consumers [34].

The most important aspect in the production of meat analogs is the correct choice of protein source. On the one hand, the choice involves the degree of availability, the yield of vegetable crops, low price, and extraction potential, while on the other hand, the balanced amino acid composition, good gelling properties, high water retention capacity, etc., are important [24]. Also, the quality of protein-rich raw materials is responsible for obtaining a fibrous structure for meat analogs, which is considered a major problem in the manufacturing process of these products [17].

The results regarding the chemical composition of meat analog samples are presented in Table 1.

As shown in Table 1, the higher temperature regime (60-80-100-120 °C) led to a slight decrease in the content of DM, PC, and CHC in the final products compared to temperature barrel 40-60-80-100 °C. This may be due to higher moisture loss after extrusion and during frozen storage. The FC and AC remained relatively stable, while the CFC slightly increased. The increase in crude fiber may result from more efficient cell wall destruction and fiber release at higher temperatures, which can affect the textural properties of the product. Control samples 1PPI and 2PPI demonstrated significantly higher PC in the final products, 37.83% and 34.49%, respectively, which is expected given the high PC in the PPI [17]. However, it was important to note that the extrusion process and the interaction of components can influence the final protein concentration. Mixtures of S, C, and HM with PPI showed lower PC in the final products. This is because meat analogs containing S (10.10%) and C (22.05%) had significantly lower protein compared to PPI [17,19]. On the other hand, HM, despite its lower PC (34.98%) compared to PPI (76.00%) [17], produced a relatively high PC in meat analogs, with 1HM:PPI—26.92% and 2HM:PPI—27.47% compared to samples that contain S and C.

The FC in meat analogs also varied depending on the blend composition. HM contributed significantly to the FC (1.23–1.45%), which is expected given its origin from oilseeds. Other samples showed relatively low fat content.

The CHC, on the other hand, was significantly higher in samples containing S and C. The results for 1S:PPI and 2S:PPI were 22.31% and 21.27%, and for 1C:PPI and 2C:PPI were 17.38% and 17.94%, respectively. This directly correlated with the high starch and other carbohydrate content in these types of raw materials [17,19]. Protein isolates, as their name suggests, contained minimal amounts of carbohydrates, which was confirmed by the low carbohydrate content in 1PPI—9.36% and 2PPI—8.18%.

The analysis showed that both the extrusion temperature regime and the composition of the raw materials had a significant effect on the physicochemical properties of meat analogs.

Protein denaturation at high temperatures can lead to the formation of new bonds and aggregates, which affect WHC and texture [19].

For mixtures with S and C, where there was a significant amount of starch, the temperature regime will also affect the degree of starch gelatinization. Gelatinized starch can act as a binding agent and affect the texture and WHC of the product. Higher temperatures (60-80-100-120) promoted more complete gelatinization of starch, which may improve the structural integrity of meat analogs [17].

The ash content in meat analogs was an important indicator reflecting the concentration of mineral substances. When comparing the ash content in raw materials and finished products, both, based on pure isolate and their mixtures, showed a decrease in ash content in the finished product compared to the initial plant protein sources [17,19]. A similar pattern was confirmed by Pennells et al. who reported a decrease in ash content after low-moisture extrusion of pea and soy proteins [35].

One reason may be the leaching of minerals: under wet extrusion conditions, some water-soluble elements (sodium, potassium) were lost with water [36,37]. On the other hand, mechanical stress and high temperatures can lead to minor losses of small product particles containing minerals during transport or drying.

When comparing temperature regimes (40–60–80–100 °C and 60–80–100–120 °C), in most mixtures, the ash content was slightly higher at higher temperatures. Thus, in 1PPI the ash content was 2.16% versus 2.21% for 2PPI. This may be due to the thermal binding of components and reduced mineral losses, as suggested by Pennells et al. [35]. However, the difference was insignificant, indicating a secondary role of temperature in mineral loss.

When analyzing the effect of extrusion on the CFC in samples based on PPI, a significant increase was observed. This can be explained by several mechanisms. First, during extrusion, especially at high temperatures and pressures, starch can retrograde, turning into resistant starch, a component classified as dietary fiber. This effect was described by Tas Ayten et al. where an increase in resistant starch levels up to 3.2% was observed in extrudates based on pea flour [38]. This may also be due to the degradation of heat-sensitive fibers at high extrusion temperatures. Pennells et al. explicitly stated that fibers present in isolates can be broken down into fractions that cannot be determined by fiber analysis methods [35].

In mixtures with PPI, a decrease in fiber was observed compared to the original S and C [17,18]. In mixtures with HM, an increase was observed compared to 1PPI and 2PPI. This effect can be explained by the simultaneous degradation of fibers from flour and the formation of new structures (e.g., resistant starch) from PPI. A similar combined effect has been described by Snel et al. where reprocessing of proteins showed changes in available fiber depending on the composition of the mixture [36].

pH values for meat analogs are an important quality indicator in identifying appropriate storage conditions, considering the susceptibility of these products to storage due to a high moisture content.

In general, meat analogs had a pH close to neutral values, compared to meat, which is more acidic. However, the pH value was greatly influenced by the type of raw materials used. The results obtained showed high values for the reference sample 1PPI and 2PPI and the lowest values 6.63 for the 1HM:PPI and 5.96 for the 2HM:PPI meat analog samples. For the meat analogs samples 1S:PPI, 2S:PPI and 1C:PPI, 2C:PPI, the results showed values slightly below 7.

Similar results have been presented by other authors for meat analogs from plant-based sources rich in protein. The pH of these foods was closer to 7 (vegan meatball). High water content and protein raw material water activity promotes bacterial proliferation [39]. In this context, to preserve the microbiological stability of these products, short-term storage by refrigeration or long-term storage by freezing was mandatory.

Water retention in plant proteins is influenced by protein solubility and could change depending on pH values. These proteins showed the lowest water retention at their isoelectric point due to significant protein–protein interactions through hydrophobic forces. Protein–lipid interaction occurred between the nonpolar side chains of proteins and the aliphatic chains of lipids through hydrophobic interactions. WHC and OHC are key functional properties of proteins used in meat substitute manufacturing to achieve acceptable organoleptic characteristics in the final products [40]. As shown in Table 1, the WHC and OHC had the same trend. Maximum values were obtained by the 1HM:PPI and 2HM:PPI sample, followed by 1C:PPI, 1C:PPI and 1S:PPI, 2S:PPI, with insignificant variations. In the case of extrusion at higher temperatures, the holding capacity was slightly higher. The minimum values were obtained for control samples. Some authors have suggested that WHC as well as OHC may be directly affected by the structure of meat analogs that form intra- and inter-hydrogen bonds caused by hydroxyl groups [41,42].

The AA in the hydroethanolic extracts of the prepared meat analogs was determined, and the free radical cation scavenging capacity ABTS + was expressed in mg TE/g DW. In the 1PPI, a higher AA was recorded—0.74 mg TE/g DW—compared to the values of 0.69 mg TE/g DW in the 2PPI. In the hydroethanolic extract of PPI as raw material, an AA of 2.7 mg TE/g DW was previously determined [17]. Bibliographic data attest a high content of polyphenols in pea flour, especially ferulic acid (1.22 mg/g DW), known for its pronounced antioxidant effect [43]. It has also been reported that ferulic acid is heat-sensitive; it degrades under the influence of temperatures and in unfavorable conditions [44].

The recorded data indicates that the 2C:PPI, 1HM:PPI, and 2HM:PP had the effect of increasing AA compared to the control samples. HM had the most notable contribution to increasing the biological value of the product in both barrel temperature regimes, by 70.7% and 33.8%, respectively. The AA of HM was mentioned in the previous study by Bulgaru et al. with values of 4.09 mg TE/g DW [17]. These are due to the rich content of polyphenols, including flavonoids and dimers, trimers, and tetramers of procyanidins [45], as well as volatile compounds, vitamins, and amino acids [46].

For the 2C:PPI samples, compared to the control, AA increased by 29.0%. This can be explained by the depolymerization of procyanidin macromolecules at high temperatures and the release of dimers, trimers, and monomers with antioxidant effects. Also, at higher temperatures, polypeptide chains are more intensively cleaved into amino acids with antioxidant properties, such as cysteine, methionine, and lysine. Bulgaru et al. determined AA by ABTS in chickpea flour hydroethanolic extracts of 0.92 mg TE/g DW [17]. These data were comparable to those reported by Constantini et al. for hydromethanolic extract (20%) of chickpea flour, which does not exceed 0.68 mg TE/g DW [43].

### 3.2. Texture Profile Analysis of Meat Analogs

The textural parameters assessed by texture profile analysis are influenced by factors such as protein type and concentration, lipid content, and processing temperature. The results of the texture profile analysis are presented in Table 2.

Hardness is a critical textural property of meat analogs, as it directly influences chewiness [47]. The observed increase in hardness for PPI with higher extrusion temperatures can be attributed to protein denaturation and aggregation, which lead to a firmer structure [48]. However, the incorporation of S, C, and HM into PPI resulted in lower hardness overall, with the highest hardness recorded in 2HM:PPI. Additionally, differences in hardness across formulations can be attributed to variations in fiber content, as well as differences in the water solubility and water-holding capacities of proteins. Resilience is defined as a product’s ability to regain its original height after compression. Higher resilience suggests a firmer network structure that snaps back quickly, mimicking the bite and juiciness. Resilience was significantly affected by protein composition and temperature. Chewiness, which measures the effort required to chew a product before swallowing [49], exhibited a trend similar to that of hardness, as these properties are inherently related [50]. The incorporation of S, C, and HM into PPI led to lower chewiness values at both temperatures. A high chewiness value indicates a denser and firmer texture, requiring more force to chew before swallowing. The texture required to imitate meat by meat analogs is desired to have higher hardness and chewiness. In order to have optimum consumer acceptance, these textural characteristics should be very similar to those found in conventional meat products [51].

### 3.3. Color Measurement of Meat Analogs

The color parameters of the raw materials played a fundamental role in determining the final color of extruded products. As can be seen from Table 3, PPI was characterized by a moderate BI (30.10) and C* (20.55). The h* (79.60), close to 90°, indicated a predominance of yellow tones, which was consistent with its light-yellow appearance (L* = 84.32, b* = 20.21). This is typical for plant proteins, where natural pigments or oxidation products may be present. The S had the lowest BI (17.63) and C* (13.63). This indicated its lowest tendency to darken and its least saturated color. The h* (83.64°) was also in the yellow range, but with lower intensity, which corresponded to its L* (87.93) and low a* and b* values (1.51 and 13.55, respectively). The C had moderate BI (21.53) and C* (17.33) values. Although it was the lightest component (L* = 91.76), its h* (85.33°) indicated a pronounced yellow hue, which may be due to the natural pigments of the chickpea. This suggested that, although the initial color was light, there was potential for darkening reactions during processing. HM stood out sharply, with the highest BI (58.37) and C* (23.14). This indicated its intense, saturated, and already significantly darkened color in its original state. The h* (66.33°) was shifted towards orange-red tones. The initial color characteristics of the raw materials were the starting point for understanding how extrusion and mixing of different components will affect the final color of meat analogs.

A significant change in lightness (L*) values was detected across all samples, except HM:PPI samples, when the process temperature profile was raised from 40-60-80-100 °C to 60-80-100-120 °C, signifying that the products appeared darker. Moreover, blending equal quantities of S and C with PPI produced lighter-hued meat analogs, whereas the inclusion of HM resulted in darker-hued meat analogs. Color assessments of the raw ingredients indicated that S and C exhibited a lighter shade compared to PPI, while HM was noted to be darker. As anticipated, the color characteristics of meat analogs are influenced by the color of the raw materials and the processing temperature.

The Maillard reaction is the main factor driving color changes during processing [52]. Amino acid cross-linking in the presence of reducing sugars contributes to color and flavor development, influenced by extrusion parameters and raw material composition [53]. A trend of decreasing lightness with increasing temperature was observed, as the Maillard reaction and caramelization accelerate under high temperature and high protein concentration, leading to a darker color [48]. Therefore, increasing the temperature profile from 40-60-80-100 °C to 60-80-100-120 °C resulted in darker-colored products. Additionally, due to the characteristic colors of raw materials, blended products exhibited different lightness values compared to the control group. Since S and C were lighter in color than PPI, their addition resulted in lighter-colored meat analogs compared to the control samples. On the other hand, the characteristic dark color of HM led to noticeably darker meat analogs, and as a result, no significant lightness difference was observed in 1HM:PPI and 2HM:PPI products. Regarding redness, differences were observed between blended products and the control, rather than between temperature levels. The addition of S, C, and HM to PPI at both temperatures led to a decrease in redness values, primarily due to the characteristic colors of the raw materials. Color measurements of the individual ingredients confirmed that all raw materials had lower redness values compared to PPI, except HM. According to Ramos Diaz et al. [52], redness is influenced by compositional aspects. Except for HM-containing products, yellowness values varied with increasing temperature, particularly in control and blended formulations. This can also be attributed to the characteristic colors of the raw materials and their blending effects.

BI increased significantly for 1PPI and 2PPI samples due to extrusion by over 79.7 and 94.8%, respectively, confirming intense darkening. Studies showed that as the temperature rises, the color of extruded products made from pea protein, which contains sufficient amounts of lysine and other amino acids that promote Maillard reactions, usually darkens [54,55]. C* changed insignificantly—increasing by 8.7% under a milder extrusion regime (1PPI), indicating a more saturated color, and decreasing by 16% under a harsher regime (2PPI). This may be due to the fact that with very intense darkening, the color becomes duller despite its darkness. This phenomenon may be due to the formation of large amounts of melanoidins, which can absorb light in a wide spectrum, reducing color purity. The h* decreased by an average of 13% for 1PPI and 14% for 2PPI, shifting towards orange-red tones, which corresponded to the appearance of brown hues characteristic of Maillard reaction products [56].

The addition of S led to obtaining meat analog samples with a lower BI of 37.68 for 1S:PPI and 45.63 for 2S:PPI compared to the control sample. This was due to the low initial BI of S (17.63) and its ability to dilute the concentration of reactive PPI components. S may also contain antioxidants that slow down darkening reactions [57]. The increase in C* at higher temperatures for these samples may be related to pigment concentration or the formation of new chromophores, which impart a brighter hue but not necessarily a darker one. The shift in h* towards yellow hues confirmed the influence of the original S color.

Despite its initial lightness, C significantly increased the BI of extrudates, indicating its active participation in darkening reactions. Chickpeas are rich in carbohydrates and proteins, which are substrates for Maillard and caramelization reactions [58]. A significant increase in C* and a shift in h* towards orange-yellow tones confirmed the formation of intense yellow-brown pigments. This may be due to the high content of sugars and amino acids in C, which form melanoidins and other darkening products during extrusion.

HM, being initially dark and saturated, led to the darkest products (low L* 33.57 for 1:HM:PPI and 34.15 for 2HM:PPI), but had relatively low BI after extrusion compared to other mixtures. This may be because most of the darkening reactions have already occurred during the nut processing, and further extrusion does not lead to significant additional formation of brown pigments, but rather to the degradation of existing ones. A sharp decrease in C* (comparing the values of HM-raw material and samples of meat analogs containing HM) indicated a loss of brightness and color purity, which may be the result of the original shades being masked by intense darkening and the formation of dull, neutral pigments. The shift of h* towards red tones indicated the formation of more reddish dark brown pigments, different from the original yellow-brown pigments of hazelnut. This was consistent with studies showing that heat treatment can lead to pigment degradation and the formation of new compounds that affect color [59].

Analyzing the effect of extrusion temperature conditions on BI, C*, and h*, it can be seen that, in general, an increase in extrusion temperature led to an increase in BI, which was to be expected, since the Maillard reaction and caramelization are accelerated at higher temperatures [60]. However, in 1PPI and 2 PPI, a slight decrease in BI is observed under a more severe regime (60-80-100-120 °C) compared to a milder one (40-60-80-100 °C). This may be because at very high temperatures and short residence times, some intermediate products of the Maillard reactions may degrade or polymerize into compounds that have less effect on BI, or the pigments may degrade more rapidly.

For mixtures with S and C, the increase in temperature consistently increased BI, confirming their sensitivity to thermal effects.

The behavior of C* during extrusion was ambiguous and strongly depended on the type of raw material. For samples 1PPI and 2PPI, C* first increased and then decreased under a more severe regime, indicating a loss of brightness with excessive darkening. For mixtures with S and C, C* generally increased with increasing temperature, indicating an increase in color saturation for samples 2S:PPI and 2C:PPI. However, for mixtures with HM, C* decreased significantly after extrusion and remains low, indicating dull formation and less vivid color. This emphasizes that C* did not always correlate with BI, and intense darkening did not always mean a more saturated color; sometimes it can lead to the formation of a dull, less expressive color.

h* decreased consistently for meat analogs 1PPI, 2PPI, and mixtures with C and HM compared to raw materials, indicating a shift in color tone from yellow to orange-red. This was typical of Maillard reactions, which formed brown pigments with a reddish tint [56]. For the mixture with S, h* remained relatively high and even increased slightly under a more severe regime, which may be due to the stability of S yellow pigments or the specificity of darkening reactions in its presence.

The ΔE* parameter, a dimensionless value calculated from the L*, a*, and b* coordinates, serves as an indicator of perceptible color differences in meat analog samples as detected by the human eye. Analyzing the results obtained for the ΔE parameter after the classification of the color difference shown by Lo Faro et al. [61], all the meat analog samples presented ΔE* values > 12, which indicated completely different colors, easily perceived by the human eye (Table 4). An obvious increase in the ΔE* parameter with increasing barrel temperature profile was observed for the 2S:PPI and 2C:PPI samples. For the meat analog containing HM, a decrease in the ΔE* value was observed but still fell into the ΔE* > 12 categories. The impact of raw material color on the final product was evident, alongside chemical changes occurring during extrusion, such as Maillard reaction, caramelization, hydrolysis, and nonenzymatic pigment degradation, which significantly contributed to color changes [49].

### 3.4. Protein Digestibility of Meat Analogs

**Figure 1:**
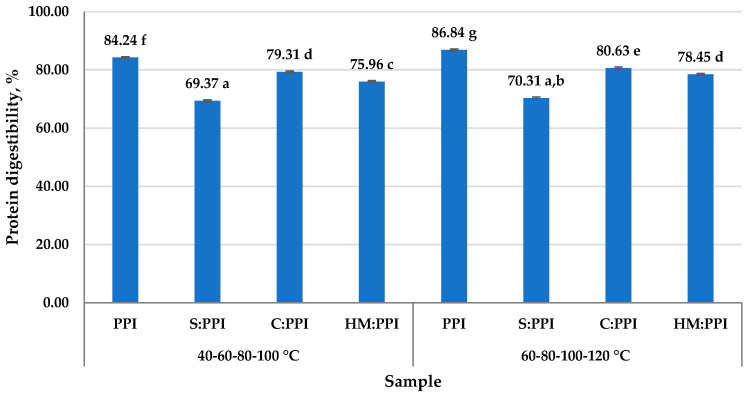
Protein digestibility of high moisture meat analogs. The results are mean ± standard deviation; different letters (^a–g^) designate statistically different results (*p* ≤ 0.05).

Plant-based meat analogs are known as foods that have a low digestibility, 40–90%. The main factors influencing the degree of digestibility are the bioavailability of contained chemical compounds, such as proteins, lipids, and starch, as well as the modulation of the microstructure of plant nutrients [62]. Previous research has shown that the increased particle size, along with the excessive use of binding agents, hinders the interaction between digestive enzymes and the food matrix in plant-based meat analogs, which leads to lower digestibility [63]. The results regarding the protein digestibility of the analyzed meat analogs are presented in Figure 1.

1PPI and 2PPI samples showed the highest digestibility values, possibly due to its complete essential amino acid profile [17]. Some authors reported similar values for pea protein isolate ranging from 80 to 85% [64]. Higher extrusion temperatures, up to 120 °C, slightly increase digestibility, indicating a slight denaturation of the proteins.

Meat analogs obtained by mixing S, C, and HM with PPI showed a lower digestibility than samples manufactured from only PPI. Intermediate values were obtained for the meat analog samples: 79.31% for 1C:PPI, 80.63% for 2C:PPI, and 75.96% for 1HM:PPI; 78.45%—2HM:PPI. A lower digestibility of about 69.37% and 70.31% was obtained for 1S:PPI and 2S:PPI samples, respectively. According to previous studies, obtaining such results was impacted by low solubility and high molecular weight of plant proteins [65], higher viscosity, which can reduce the reaction rate by affecting the contact between proteins and digestive enzymes, the presence of antinutritional substances, trypsin inhibitors, the presence of insoluble fiber, and their starch content [66].

Heat treatment performed at higher final temperatures, up to 120 °C, insignificantly improved protein digestibility. Moderate extrusion temperatures can improve protein digestibility by modifying their structure, disrupting intramolecular interactions (hydrogen bonds and hydrophobic interactions) within protein molecules, and creating structures that are more accessible to digestive enzymes [67]. Also, moderate extrusion temperatures had a positive impact on reducing antinutrients in plant raw materials, which had a positive impact on the protein digestibility. It has been demonstrated that the use of higher temperatures during extrusion can reduce digestibility if they lead to non-enzymatic browning, the formation of Maillard compounds [68].

### 3.5. Antioxidant Activity Before and After In Vitro Digestion of Meat Analogs

**Figure 2:**
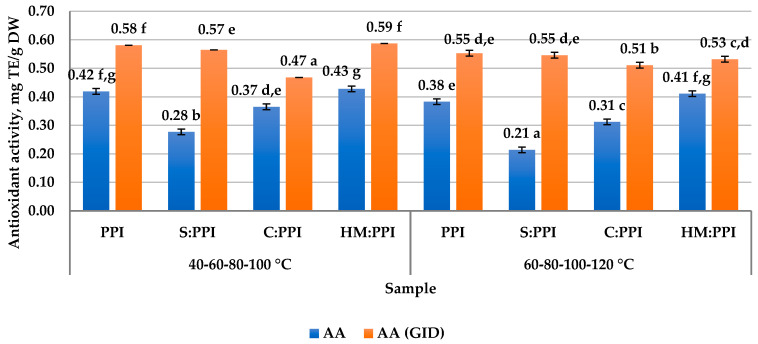
Antioxidant activity (ABTS) of meat analogs before (AA) and after in vitro digestion (AA (GID)). The results are mean ± standard deviation; different letters (^a–g^) designate statistically different results (*p* ≤ 0.05).

The analysis of the antioxidant activity of meat analogs and samples taken during the gastric and intestinal digestion (GID) phases was performed using the ABTS method, the free radical cation that functions in both hydrophilic and lipophilic environments. For the prepared meat analogs, AA was determined in aqueous extracts (Figure 2).

The aqueous solution showed values of AA between 0.428 and 0.214 mg TE/g DW; amino acids, soluble peptides, and a smaller amount of polyphenols, which are less soluble in water, were extracted. The data for the aqueous solution were compared with the data determined for the samples taken in the GID phases. The results showed an increase in AA in the GID phases compared with the result obtained before GID.

Gallego et al. reported an increase in the GID phase of AA in the ABTS test of cooked legume pastes, namely soybean (*Glycine max*) over 2-fold; lentils (*Lens cutinaris*) over 10-fold; and peas (*Pisum sativum*) over 3-fold [69].

Several studies have reported that although the conditions of in vitro food antioxidant capacity testing may not correspond to in vivo conditions, protein AA increases as a result of simulated gastrointestinal digestion [70]. The action of gastrointestinal enzymes leads to the breakdown of proteins and peptides and the formation of dipeptides, tripeptides, and free amino acids, thus affecting the antioxidant capacity. It has been reported that most antioxidant peptides formed from proteins contain between three and six amino acid residues [71].

Kut et al. reported that the release of amino acids tyrosine, tryptophan, cysteine, histidine, arginine, and cystine during denaturation and digestion increased the decolorization capacity in the ABTS assay, and cysteine, tryptophan, tyrosine and, cystine increased AA in the FRAP assay. Also, five amino acids showed reactivity in the ABTS assay in the following order: tyrosine > tryptophan > cysteine > histidine > arginine [72].

Also, at an acidic pH, amino acids will have a low dissociation rate, and the groups responsible for the antioxidant effect in amino acids and in the ABTS free radical cation will be protonated [73].

1HM:PPI and 2HM:PPI samples until GID had a higher AA compared to the rest of samples, which is presumably due to the notable antioxidant properties of hazelnut meal, which depend on the increased content of polyphenols, volatile compounds, and vitamins [48].

Several studies have reported that the antioxidant capacity of plant-based foods, particularly those attributed to polyphenols, may decline following simulated gastrointestinal digestion [74].

Simulated in vitro digestion of 36 Brazilian foods items led to an increase in the antioxidant capacity of cereals, legumes, vegetables, chocolate, and fruits, while a decrease was observed for red wine, coffee, and yerba mate throughout the digestion process [75]. This was explained by the fact that polysaccharides and proteins, under the action of digestive media, split into monosaccharides and amino acids with more pronounced reducing properties, and a series of phenolic compounds can form insoluble compounds with peptides and amino acids, or are lost along the gastrointestinal tract [76]. From the data represented in Figure 2, it could be concluded that the increase in AA in meat analog samples is explained by the digestibility of the contained proteins, which were easily broken down into amino acids in the GID process.

### 3.6. Relationship Between Physicochemical Characteristics, Protein Digestibility, Antioxidant Activity, Texture Profile Analysis, and CIELab Color Parameters in High Moisture Meat Analogs

**Figure 3:**
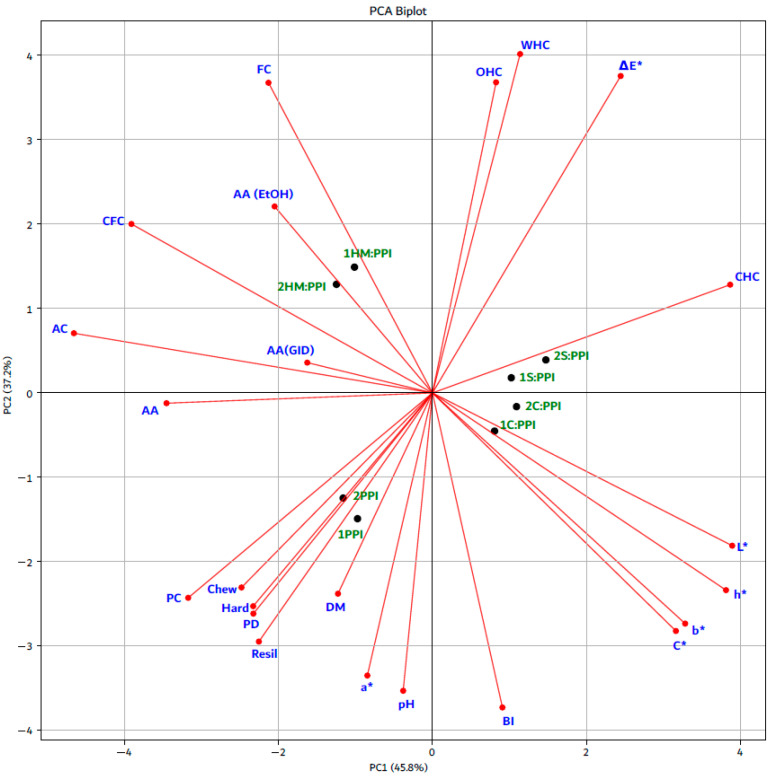
Illustrating the relationships between physicochemical characteristics, protein digestibility, antioxidant activity, texture profile analysis, and CIELab color parameters in high-moisture meat analogs formulated from different blends and subjected to two heating temperature profiles by PCA: DM—dry matter; PC—protein content; FC—fat content; AC—ash content; CFC—crude fiber content; CHC—carbohydrate content; pH—active acidity; PD—protein digestibility; WHC—water holding capacity; OHC—oil holding capacity; AA—antioxidant activity before in vitro digestion; AA (GID)—antioxidant activity after in vitro digestion; AA (EtOH)— ABTS in 70% EtOH extract; Hard—hardness; Resil—resilience; Chew—chewiness; L*—lightness; a*—redness; b*—yellowness; C*—chroma; h*—hue angle; ΔE*—total color difference; BI—browning index; PPI—pea protein isolate; S—soryz flour; C—chickpea flour; HM—hazelnut meal; 1—40-60-80-100 °C heating temperature profile; 2—60-80-100-120 °C heating temperature profile.

PCA was applied to show the relationships between physicochemical characteristics, protein digestibility, antioxidant activity, texture profile analysis, and CIELab color parameters in high-moisture meat analogs formulated from different blends (PPI, S:PPI, C:PPI, and HM:PPI) and subjected to two heating temperature profiles: 1 (40-60-80-100 °C) and 2 (60-80-100-120 °C). Figure 3 presents the scaled PCA loadings to improve visual interpretability, and the full PCA results are provided in Table S1. For each variable, the mean of three sample measurements was used, and all values were scaled to the range [0,1][0,1][0,1] using min–max scaling before performing PCA. The first principal component (PC1) and the second principal component (PC2) accounted for 45.8% and 37.2% of the total variance. PC1 was closely associated with AA, AA (GID), AC, CFC, CHC, L*, and h*, whereas PC2 was closely associated with pH, a*, DM, Resil, PD, Hard, Chew, PC, BI, C*, b*, AA (EtOH), FC, OHC, WHC, and ΔE*.

According to the PCA plot, the temperature profile showed no significant influence on the measured parameters, whereas the type of blend had a pronounced effect on sample differentiation. Specifically, 1PPI and 2PPI were positioned in the bottom-center region and were strongly associated with PC, Chew, Hard, PD, Resil, and DM. In contrast, 1S:PPI and 2S:PPI, along with 1C:PPI and 2C:PPI, clustered in the center-right of the plot and were closely related to CIELab color parameters (L*, b*, h*, C*), indicating enhanced color. Meanwhile, 1HM:PPI and 2HM:PPI were located in the center top-left region, primarily associated with FC, AA (EtOH), and CFC, highlighting their superior functional and antioxidant properties. This clear separation along PC1 and PC2 suggests that blend composition, rather than thermal treatment, was the primary factor contributing to the observed differences in physicochemical and functional characteristics.

To validate the PCA findings, one-way ANOVA was used to evaluate the effects of temperature profile and blend type on the measured parameters. The results confirmed that the temperature profile had no significant effect on most features, with only DM showing a statistically significant difference (F = 6.31, *p* < 0.05). More detailed results are provided in Table S2. In contrast, the blend type had a highly significant impact on nearly all parameters, including AA (EtOH) (F = 78.08, *p* < 0.05), CFC (F = 75.97, *p* < 0.05), Hard (F = 438.21, *p* < 0.05), and L* (F = 1037.56, *p* < 0.05). (Additional details are presented in Table S3).

These results strongly support PCA findings, suggesting that variations in functional, compositional, and color-related characteristics are primarily driven by differences in blend composition rather than by thermal treatment.

## 4. Conclusions

The results of the study showed that increasing the extrusion temperature led to a slight reduction in DM, PC, and CHC in the resulting meat analogs, likely due to enhanced thermal degradation and lower moisture loss during frozen storage. PC was significantly affected by the composition of raw materials. Among the tested blends, those containing HM provided more protein than the S and C blends, reflecting its higher protein composition. FC was significantly higher in the HM-containing formulations, consistent with its origin from oilseeds. Carbohydrate levels were higher in the S and C blends, reflecting their higher native starch content. The thermal regime also influenced starch gelatinization, particularly in the S- and C-based samples. The AC of the extruded products was generally lower, likely due to the leaching of water-soluble minerals during extrusion. The pH values remained close to neutral and varied depending on the raw material, with the HM-containing samples exhibiting the lowest pH. WHC and OHC were highest in the HM blends and increased slightly with extrusion temperature, likely due to protein–lipid–water interactions and increased porosity—factors essential for product texture and stability. The C and HM blends exhibited improved AA, with the HM-containing samples showing increases of 70.7% (1HM:PPI) and 33.8% (2HM:PPI). Higher extrusion temperatures increased hardness, likely due to enhanced protein denaturation and aggregation. The S, C, and HM blends exhibited lower hardness and chewiness, which can be attributed to differences in fiber content and the distinct water retention and solubility characteristics of the composite proteins. Strength increased with temperature, reflecting improved protein crosslinking and elastic recovery, while chewiness followed a similar trend. Color parameters—L*, C*, h*, and BI—were significantly affected by both the color of the raw materials and the extrusion conditions. Higher extrusion temperatures (60–120 °C) produced darker products as a result of Maillard reactions and caramelization. PD was highest in the pure PPI formulations (~80–85%) and decreased in the S, C, and HM blends (~69–70%). Moderate extrusion temperatures slightly enhanced digestibility by reducing antinutrients and promoting partial protein denaturation. PCA effectively elucidated the relationships among physicochemical, nutritional, textural, and color properties. PC1 and PC2 accounted for 83% of the total variance, revealing strong positive correlations between PC, AA, PD, and textural parameters (hardness, chewiness, resilience), as well as inverse correlations with color parameters (L*, b*, C*, h*).

## 5. Future Outlook

Plant-based alternatives are becoming increasingly necessary as a potential response to the severe environmental and health impacts of animal-based food options. The future of plant-based meat alternatives is promising, driven by continued advances in emerging manufacturing techniques and sustainable ingredients. Thus, the results obtained outline how the findings on protein denaturation and aggregation under high-moisture extrusion can be leveraged, guiding both industrial innovation and fundamental research toward sustainable, health-oriented, and technologically advanced food systems. Integrating advanced analyzing tools such as real-time spectroscopy and rheology will illuminate the pathways of denaturation and aggregation, while proteomic and metabolomic profiling will identify off-flavor and by-product precursors. Leveraging these findings to valorize food-industry side streams and apply targeted protein-engineering approaches will accelerate the creation of ingredient blends with ideal textures and enhanced nutritional value. Finally, coordinated in vitro and in vivo assessments of digestibility, bioavailability, and inflammatory responses, together with comprehensive life-cycle analyses grounded in circular economy principles, will ensure environmental sustainability and inform regulatory frameworks for global consumer acceptance.

## Figures and Tables

**Table 1:** Physicochemical indices and AA of analyzed meat analogs.

Heating Temperature Profile, °C	Blends	DM, %	PC, %	FC, %	AC, %	CFC, %	CHC, %	pH	WHC, mL g^−1^	OHC, mL g^−1^	AA, mg TE/g DW (ABTS in 70% EtOH Extract)
40-60-80-100	1PPI	49.66 ± 0.05 ^e^	37.83 ± 0.07 ^g^	0.31 ± 0.01 ^a^	2.16 ± 0.02 ^c^	1.62 ± 0.02 ^c^	9.36 ± 0.15 ^b^	7.95 ± 0.01 ^d^	2.43 ± 0.04 ^b^	1.75 ± 0.0 ^a^	0.74 ± 0.03 ^c^
	1S:PPI	45.69 ± 0.09 ^d^	21.81 ± 0.10 ^b^	0.29 ± 0.01 ^a^	1.28 ± 0.01 ^b^	0.96 ± 0.0 ^a^	22.31 ± 0.11 ^g^	6.99 ± 0.01 ^c^	3.42 ± 0.06 ^d^	2.77 ± 0.05 ^d,e^	0.54 ± 0.02 ^a^
	1C:PPI	43.48 ± 0.07 ^c^	24.22 ± 0.09 ^c^	0.58 ± 0.02 ^b^	1.30 ± 0.01 ^b^	0.93 ± 0.01 ^a^	17.38 ± 0.08 ^d^	6.91 ± 0.01 ^c^	3.23 ± 0.05 ^c^	2.27 ± 0.06 ^c^	0.61 ± 0.04 ^a,b^
	1HM:PPI	42.88 ± 0.05 ^b^	26.92 ± 0.06 ^d^	1.23 ± 0.02 ^c^	2.34 ± 0.02 ^d^	2.03 ± 0.02 ^d^	12.39 ± 0.01 ^c^	6.63 ± 0.01 ^b^	3.88 ± 0.03 ^f^	2.69 ± 0.04 ^d^	1.27 ± 0.03 ^f^
60-80-100-120	2PPI	45.13 ± 0.08 ^d^	34.49 ± 0.11 ^f^	0.25 ± 0.01 ^a^	2.21 ± 0.01 ^c^	1.45 ± 0.01 ^b^	8.18 ± 0.09 ^a^	7.87 ± 0.01 ^d^	2.22 ± 0.02 ^a^	2.16 ± 0.03 ^b^	0.69 ± 0.02 ^b,c^
	2S:PPI	43.00 ± 0.06 ^b,c^	20.24 ± 0.08 ^a^	0.26 ± 0.01 ^a^	1.19 ± 0.01 ^b^	0.91 ± 0.01 ^a^	21.27 ± 0.12 ^f^	6.93 ± 0.01 ^c^	3.69 ± 0.05 ^e^	2.75 ± 0.04 ^d^	0.54 ± 0.03 ^a^
	2C:PPI	41.59 ± 0.04 ^a^	22.05 ± 0.09 ^b^	0.50 ± 0.02 ^b^	1.10 ± 0.01 ^a^	0.91 ± 0.02 ^a^	17.94 ± 0.09 ^e^	6.88 ± 0.01 ^c^	3.72 ± 0.04 ^e^	2.33 ± 0.02 ^c^	0.89 ± 0.03 ^d,e^
	2HM:PPI	43.27 ± 0.07 ^c^	27.47 ± 0.12 ^e^	1.45 ± 0.01 ^d^	2.44 ± 0.0 ^d^	2.28 ± 0.03 ^e^	11.91 ± 0.13 ^c^	5.96 ± 0.01 ^a^	4.09 ± 0.06 ^g^	2.89 ± 0.05 ^e^	0.92 ± 0.04 ^d,e^

DM—dry matter; PC—protein content; FC—fat content; AC—ash content; CFC—crude fiber content; CHC—carbohydrate content; pH—active acidity; WHC—water holding capacity; OHC—oil holding capacity; AA—antioxidant activity; PPI—pea protein isolate; S—soryz flour; C—chickpea flour; HM—hazelnut meal; 1—40-60-80-100 °C heating temperature profile; 2—60-80-100-120 °C heating temperature profile. The results are mean ± standard deviation; different letters (^a–g^) designate statistically different results (*p* ≤ 0.05).

**Table 2:** Texture profile analysis results of high moisture meat analogs.

Heating Temperature Profile, °C	Blends	Hardness, N	Resilience, %	Chewiness, N
40-60-80-100	1PPI	224.12 ± 17.70 ^e^	41.70 ± 0.30 ^f^	152.68 ± 25.97 ^d,e^
	1S:PPI	146.73 ± 8.78 ^b^	35.34 ± 0.57 ^e^	78.13 ± 17.12 ^b^
	1C:PPI	171.19 ± 9.59 ^c^	30.17 ± 0.66 ^c^	101.73 ± 18.04 ^b,c^
	1HM:PPI	137.85 ± 6.43 ^a,b^	24.37 ± 0.73 ^a,b^	82.19 ± 5.21 ^b^
60-80-100-120	2PPI	320.85 ± 5.91 ^f^	45.17 ± 0.67 ^g^	244.63 ± 4.58 ^f^
	2S:PPI	121.98 ± 2.92 ^a^	23.16 ± 0.93 ^a^	56.89 ± 6.77 ^a,b^
	2C:PPI	129.34 ± 4.94 ^a,b^	25.62 ± 0.80 ^b^	64.34 ± 9.54 ^a,b^
	2HM:PPI	186.73 ± 12.23 ^c,d^	34.32 ± 0.76 ^d,e^	125.21 ± 7.15 ^c,d^

PPI—pea protein isolate; S—soryz flour; C—chickpea flour; HM—hazelnut meal; 1—40-60-80-100 °C heating temperature profile; 2—60-80-100-120 °C heating temperature profile. The results are mean ± standard deviation; different letters (^a–g^) designate statistically different results (*p* ≤ 0.05).

**Table 3:** CIELab color parameters of high-moisture meat analogs.

Heating Temperature Profile, °C	Raw Materials/Blends	CIELab Color Parameters						
		L*	a*	b*	C*	h*, °	ΔE*	BI
-	PPI	84.32 ± 0.93 ^f^	3.71 ± 0.07 ^b^	20.21 ± 0.46 ^e^	20.55 ± 0.25 ^d^	79.60 ± 0.18 ^d^	-	30.10 ± 0.37 ^b^
	S	87.93 ± 1.27 ^f,g^	1.51 ± 0.30 ^a^	13.55 ± 0.53 ^b,c^	13.63 ± 0.29 ^b^	83.64 ± 0.11 ^e^	-	17.63 ± 0.18 ^a^
	C	91.76 ± 1.30 ^g^	1.41 ± 0.33 ^a^	17.27 ± 0.38 ^d^	17.33 ± 0.36 ^c^	85.33 ±0.13 ^f^	-	21.53 ± 0.27 ^a^
	HM	56.43 ± 2.20 ^c^	9.29 ± 0.08 ^f^	21.19 ± 0.53 ^e^	23.14 ± 0.26 ^e,f^	66.33 ± 0.09 ^b^	-	58.37 ± 0.31 ^e,f^
40-60-80-100	1PPI	54.15 ± 1.13 ^c^	8.19 ± 0.01 ^e^	20.77 ± 0.46 ^e^	22.33 ± 0.29 ^e^	68.48 ± 0.11 ^b^	-	58.63 ± 0.36 ^f^
	1S:PPI	71.86 ± 1.45 ^e^	4.90 ± 0.50 ^b,c^	20.34 ± 0.35 ^e^	20.92 ± 0.41 ^d,e^	76.46 ± 0.08 ^c^	18.06 ± 0.11 ^b^	37.68 ± 0.24 ^c^
	1C:PPI	66.77 ± 0.91 ^d,e^	6.17 ± 0.17 ^d^	26.01 ± 0.42 ^g^	26.73 ± 0.26 ^g^	76.66 ± 0.14 ^c^	13.87 ± 0.19 ^a^	55.10 ± 0.31 ^e^
	1HM:PPI	33.57 ± 0.97 ^a^	4.79 ± 0.30 ^b,c^	7.62 ± 0.25 ^a^	9.00 ± 0.27 ^a^	57.85 ± 0.09 ^a^	24.66 ± 0.26 ^d^	35.78 ± 0.17 ^b^
60-80-100-120	2PPI	44.68 ± 0.14 ^b^	6.08 ± 0.28 ^d^	16.15 ± 0.62 ^c,d^	17.26 ± 0.35 ^c^	69.37 ± 0.12 ^b^	-	54.09 ± 0.29 ^e^
	2S:PPI	67.93 ± 1.28 ^e^	4.63 ± 0.13 ^b,c^	23.05 ± 0.77 ^f^	23.51 ± 0.42 ^e,f^	78.64 ± 0.19 ^d^	24.36 ± 0.09 ^d^	45.63 ± 0.26 ^d^
	2C:PPI	61.97 ± 1.61 ^d^	6.35 ± 0.01 ^d^	27.30 ± 0.93 ^g,h^	28.03 ± 0.64 ^h^	76.91 ± 0.08 ^d^	20.60 ± 0.10 ^c^	64.15 ± 0.51 ^f^
	2HM:PPI	34.15 ± 0.53 ^a^	4.94 ± 0.15 ^c^	7.62 ± 0.18 ^a^	9.08 ± 0.16 ^a^	57.04 ± 0.06 ^a^	13.62 ± 0.14 ^a^	35.43 ± 0.21 ^b^

PPI—pea protein isolate; S—soryz flour; C—chickpea flour; HM—hazelnut meal; 1—40-60-80-100 °C heating temperature profile; 2—60-80-100-120 °C heating temperature profile; L*—lightness; a*—redness; b*—yellowness; C*—chroma; h*—hue angle; ΔE*—total color difference; BI—browning index. The results are mean ± standard deviation; different letters (^a–h^) designate statistically different results (*p* ≤ 0.05).

**Table 4:** Images of high-moisture meat analogs.

Heating Temperature Profile, °C	40-60-80-100				60-80-100-120			
Blends	1PPI	1S:PPI	1C:PPI	1HM:PPI	2PPI	2S:PPI	2C:PPI	2HM:PPI
	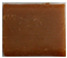	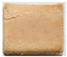	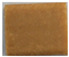	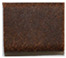	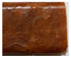	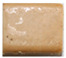	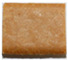	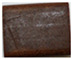

## Data Availability

The original contributions presented in the study are included in the article/Supplementary Materials. Further inquiries can be directed to the corresponding author.

## Supplementary Materials

The following supporting information can be downloaded at: https://www.mdpi.com/article/10.3390/foods14172939/s1, Table S1. PCA loadings for Physicochemical Characteristics, Protein Digestibility, Antioxidant Activity, Texture Profile Analysis, and CIELab Color Parameters in High-Moisture Meat Analogs. Table S2. ANOVA Comparisons of Different Temperature Profiles for Physicochemical Characteristics, Protein Digestibility, Antioxidant Activity, Texture Profile Analysis, and CIELab Color Parameters in High-Moisture Meat Analogs. Table S3. ANOVA Comparisons of Different Blends for Physicochemical Characteristics, Protein Digestibility, Antioxidant Activity, Texture Profile Analysis, and CIELab Color Parameters in High-Moisture Meat Analogs.
