# Supplementary material for: Qualitative and Antioxidant Evaluation of High-Moisture Plant-Based Meat Analogs Obtained by Extrusion
Source: Foods. 2025 Aug 23;14(17):2939. doi: 10.3390/foods14172939 (PMC12427645; doi:10.3390/foods14172939)
Supplement: Supplementary file 1 [file foods-14-02939-s001.zip › Table S1.pdf]

Table S1. PCA loadings for Physicochemical Characteristics, Protein Digestibility, Antioxidant Activity, Texture Profile Analysis, and CIELab Color Parameters in High-Moisture Meat Analogs

Feature	PC1	PC2
AA	0.260	-0.017
AA (EtOH)	0.150	0.148
AA(GID)	0.076	0.098
AC	0.384	0.022
BI	-0.077	-0.289
C*	-0.251	-0.216
CFC	0.293	0.112
CHC	-0.295	0.115
Chew	0.184	-0.186
DM	0.083	-0.181
FC	0.191	0.269
Hard	0.171	-0.201
L*	-0.315	-0.127
OHC	-0.007	0.280
PC	0.236	-0.206
PD	0.172	-0.218
Resil	0.169	-0.237
WHC	-0.066	0.301
a*	0.061	-0.251
b*	-0.256	-0.210
h*	-0.298	-0.170
pH	0.015	-0.274
ΔE*	-0.180	0.300
