# Supplementary material for: Qualitative and Antioxidant Evaluation of High-Moisture Plant-Based Meat Analogs Obtained by Extrusion
Source: Foods. 2025 Aug 23;14(17):2939. doi: 10.3390/foods14172939 (PMC12427645; doi:10.3390/foods14172939)
Supplement: Supplementary file 1 [file foods-14-02939-s001.zip › Table S2.pdf]

Table S2. ANOVA Comparisons of Different Temperature Profiles for Physicochemical Characteristics, Protein Digestibility, Antioxidant Activity, Texture Profile Analysis, and CIELab Color Parameters in High-Moisture Meat Analogs

Feature	F-statistic	p-value	R^2
AA	3.26	0.084	0.128
AA (EtOH)	0.19	0.663	0.008
AA (GID)	0.86	0.363	0.037
AC	0.01	0.910	0.001
BI	0.41	0.526	0.018
C*	0.02	0.879	0.001
CFC	0.01	0.917	0.001
CHC	0.06	0.809	0.002
Chew	0.60	0.448	0.026
**DM**	**6.31**	**0.019**	**0.222**
FC	0.01	0.933	0.000
Hard	0.90	0.354	0.040
L*	0.64	0.431	0.028
OHC	1.33	0.261	0.056
PC	0.41	0.526	0.018
PD	0.46	0.504	0.020
Resil	0.08	0.781	0.003
WHC	0.25	0.620	0.011
a*	0.84	0.368	0.037
b*	0.01	0.918	0.000
h*	0.10	0.749	0.004
pH	0.89	0.356	0.038
ΔE*	0.01	0.936	0.001
