# Supplementary material for: Qualitative and Antioxidant Evaluation of High-Moisture Plant-Based Meat Analogs Obtained by Extrusion
Source: Foods. 2025 Aug 23;14(17):2939. doi: 10.3390/foods14172939 (PMC12427645; doi:10.3390/foods14172939)
Supplement: Supplementary file 1 [file foods-14-02939-s001.zip › Table S3.pdf]

Table S3. ANOVA Comparisons of Different Blends for Physicochemical Characteristics, Protein Digestibility, Antioxidant Activity, Texture Profile Analysis, and CIELab Color Parameters in High-Moisture Meat Analogs

Feature	F-statistic	p-value	R^2
**AA**	10.58	3.697×10^{-3}	0.799
**AA (EtOH)**	78.08	2.889×10^{-6}	0.967
AA (GID)	3.46	7.138×10^{-2}	0.564
**AC**	150.61	2.239×10^{-7}	0.983
**BI**	331.22	9.982×10^{-9}	0.992
**C***	354.46	7.628×10^{-9}	0.993
**CFC**	75.97	3.210×10^{-6}	0.966
**CHC**	772.09	3.449×10^{-10}	0.997
**Chew**	691.57	5.349×10^{-10}	0.996
**DM**	164.80	1.572×10^{-7}	0.984
**FC**	210.75	5.968×10^{-8}	0.988
**Hard**	438.21	3.286×10^{-9}	0.994
**L***	1037.56	1.062×10^{-10}	0.997
**OHC**	8.71	6.701×10^{-3}	0.766
**PC**	716.98	4.633×10^{-10}	0.996
**PD**	22.77	2.844×10^{-4}	0.895
**Resil**	93.18	1.458×10^{-6}	0.972
**WHC**	24.51	2.190×10^{-4}	0.902
**a***	46.07	2.158×10^{-5}	0.945
**b***	461.88	2.666×10^{-9}	0.994
**h***	167.92	1.460×10^{-7}	0.984
**pH**	238.06	3.689×10^{-8}	0.989
**ΔE***	472.55	2.435×10^{-9}	0.994
